# Managing ECT related cognitive side effects: An individual approach

**DOI:** 10.1192/j.eurpsy.2021.86

**Published:** 2021-08-13

**Authors:** E. Verwijk

**Affiliations:** Brain And Cognition, University of Amsterdam, Amsterdam, Netherlands

**Keywords:** individual variablity, Depression, Cognitive side effects, pre-treatment predictors

## Abstract

Electroconvulsive brain stimulation may represent the strongest manipulation available to study brain plasticity in humans. Brain plasticity induced by electroconvulsive brain stimulation, profoundly improves disturbed emotion and motivation in patients with depression. Electroconvulsive therapy (ECT) is a highly effective and safe treatment for psychiatric disorders like severe depression. However, there is ongoing concern about the negative impact of ECT on brain function and cognition that is, surprisingly, only seen in a part of the treated patients. After 80 years of research on ECT, virtually nothing is known about the mechanisms underlying these strong individual differences in cognitive changes induced by ECT. A first step would be to better quantify the pattern and severity of the adverse cognitive outcomes in order to better distinguish patients that suffer from adverse cognitive outcomes from those that do not or even improve. By better distinguishing of these subgroups, a second step towards understanding can be taken: to identify the factors that predict adverse cognitive outcomes. Our research aims to advance understanding of the mechanisms of cognitive plasticity and reveal the pre-treatment profiles that render a patient cognitive vulnerable or resilient.

**Disclosure:**

No significant relationships.

